# 1/*x* power-law in a close proximity of the Bak–Tang–Wiesenfeld sandpile

**DOI:** 10.1038/s41598-021-97592-x

**Published:** 2021-09-13

**Authors:** Alexander Shapoval, Boris Shapoval, Mikhail Shnirman

**Affiliations:** 1 HSE University, Myasnitskaya str. 20, Moscow, Russia 101000; 2 University of Colorado Boulder, Boulder, USA; 3 Institute of Earthquake Prediction Theory and Mathematical Geophysics RAS, Profsoyuznaya 84/32, Moscow, Russia 117997

**Keywords:** Condensed-matter physics, Statistical physics, thermodynamics and nonlinear dynamics

## Abstract

A cellular automaton constructed by Bak, Tang, and Wiesenfeld (BTW) in 1987 to explain the 1/*f* noise was recognized by the community for the theoretical foundations of self-organized criticality (SOC). Their conceptual work gave rise to various scientific areas in statistical physics, mathematics, and applied fields. The BTW core principles are based on steady slow loading and an instant huge stress-release. Advanced models, extensively developed far beyond the foundations for 34 years to successfully explain SOC in real-life processes, still failed to generate truncated 1/*x* probability distributions. This is done here through returning to the original BTW model and establishing its larger potential than the state-of-the-art expects. We establish that clustering of the events in space and time together with the core principles revealed by BTW lead to approximately 1/*x* power-law in the size-frequency distribution of model events.

## Introduction

Bak, Tang, and Wisenfeld wrote “we believe that the new concept of self-organized criticality can be taken much further and might be the underlying concept for temporal and spatial scaling in a wide class of dissipative systems with extended degrees of freedom”^1^. SOC-systems evolve to a critical state characterized by power-laws without parameter tuning. The absence of adjustable parameters such as the temperature or magnetization distinguishes the SOC systems from the systems which generate the critical dynamics at the phase transition. As BTW projected, a huge quantity of real systems and processes exhibiting SOC were exposed^2–5^. Nevertheless, the power-law exponents usually depend on the features of sub-systems (f. e., seismic faults, geographical regions, forest fires, and stellar flares, respectively^6–8^), thus leaving the question regarding the extent to which the underlying systems are self-organized to be open.

The BTW model is defined on a square lattice that contains integers interpreted as grains. Initially, all lattice cells contain less than 4 grains. At each time moment a grain is added to a randomly chosen lattice cell. If the resulting number of grains is still less than 4, nothing more happens at this time moment. Otherwise, the overloaded cell transfers 4 grains in such a way that all adjacent cells (their number is 4 inside the lattice) receive 1 grain. Grains are lost off the edge of the lattice since the boundary cells do not have 4 nearest neighbors. As a result of the transfer, other cells can be overloaded. The transfers continue while there are overloaded cells. The sequence of the transfers occurred at a single time moment forms an avalanche, the size of which is the number of the transfers. For any initial distribution of grains over the lattice, the system attains a critical state characterized by the power-law size-frequency relationship of the avalanches with the exponent $\tau \approx 1.20$^9^.

Modeling of real-life systems characterized by power-laws at the critical state can be potentially performed with modifications of the original BTW model that involve various ways of stress propagation including its directed transportation, quenched disorder, and remote transfers^10–13^ and implement the BTW mechanism on different spaces including fractals and networks^14–17^. The value of the exponent $\tau$ characterizing the power-law segment $x^{-\tau }$ of the size-frequency relationship has been obtained numerically for various models; rigorous proofs have been obtained for some of them^9^.

Changes in the details of the steady loading or transport mechanism conserve the exponent $\tau \approx 1.20$, known^18^ for the BTW sandpile, for its deterministic isotropic modifications^19^. A turn to stochastic transport in isotropic sandpiles switches the exponent to $\tau \approx 1.27$^19–21^. The nature of self-organized criticality is captured by the independence of the power-laws on model details and the existence of just a few exponents within a broad class of isotropic sandpiles on the square lattice. This imposing feature of the isotropic sandpiles, nevertheless, reduces the range of its direct applications to real systems because the latter exhibit various power-law exponents.

The purpose of this paper is a BTW-mechanism extension that allows to tune the power-law exponent and belongs to a “narrow neighborhood” of the original BTW sandpile, thus compromising between a certain refusal from self-organization and keeping the mechanism staying behind it. With applications in mind, we weaken the complete separation of the slow and quick times scales, understood as the idealization of the BTW mechanism, and combine close in space and time events into mega-events. Our design of the isotropic BTW mechanism on the square lattice will lead to $\sim 1/x$ size-frequency relationship.

## Results

### Model

As in the original BTW model, we define the model dynamics on a square lattice. The cells of the lattice are numbered from 1 to $A = L^2$, where $L \in {\mathbb {N}}$ is the lattice length. Each non-boundary cell *i* shares a common side with 4 adjacent cells. These adjacent cells form the set ${\mathscr {N}}_i$ of the neighbors of the cell *i*. The boundary cells have 3 or 2 (in the case of the corner) neighbors. For any cell *i* an integer $h_i$ interpreted as the number of grains is assigned to it. A cell *i* is stable if its height $h_i < H$, where $H = 4$ is a threshold. The dynamics are given by the following procedure.

### Avalanches and their size

At each time moment $N= N_L \sim \log L$ different cells $i_1$, $\ldots$, $i_N$ are chosen at random. Their heights are increased by 1:

1 $$\begin{aligned} h_i \longrightarrow h_i + 1, \quad \forall i \in \{i_1, \ldots , i_N\}. \end{aligned}$$

If none of them attains the threshold *H*, nothing more occurs at this time moment. If at least a single height attains the threshold *H*, the grain transport starts: unstable cells pass *H* grains equally to the neighbors. Formally, for any *i* with $h_i = H$,

2 $$\begin{aligned} h_i \longrightarrow h_i - H \end{aligned}$$

3 $$\begin{aligned} h_{j} \longrightarrow h_{j} + 1 \quad \forall j \in {\mathscr {N}}_i. \end{aligned}$$

As the number of the neighbors $|{\mathscr {N}}_i|$ is 4 for the inner cell *i* and less than 4 for the boundary cell, the grain transfer (2), (3) is conservative inside the lattice and dissipative at the boundary. Let us say that each unstable cell generates an avalanche. If *n* unstable cells $\{i_1,\ldots ,i_n\}$, $n \le N$, appear as a result of the grain adding at the time *t*, then *n* avalanches $a_{i_1,t}$, $\ldots$, $a_{i_n,t}$ occur at *t*. At the beginning, each avalanche $a_{i_k,t}$, $k=1,\ldots ,n$, “propagates” to a single cell, namely, the origin $i_k$ that generates the avalanche. The size $s_{i_k,t}$ of each avalanche is set to 0 at this moment. The unstable cells $i_1$, $\ldots$, $i_n$ and their neighbors update the heights in line with (2), (3) simultaneously. The size $s_{i_k,t}$ of the avalanches $a_{i_k,t}$ is increased from 0 to 1. The updates can induce instability in other cells. New unstable cells are associated with just those avalanches that propagate to them. In other words, if an unstable cell *j* obtained a grain from a cell $j'$ associated with the avalanche $a_{i,k}$, then *j* is also associated with $a_{i,k}$. If two (or more) avalanches propagate to *j* (i. e., pass a grain to *j*) simultaneously, then the choice of the avalanche to be assigned to *j* is performed at random. Each update induced by the instability of the cell associated with the avalanche $a_{i_k,t}$ results in the rise of its size $s_{i_k, t}$ by 1, $k = 1,\ldots , n$. The updates ruled by (2) and (3) occur while there are unstable cells. As soon as $h_i < H$ for all cells *i*, the next time moment begins.

Note that a cell can attain the threshold *H* several times within a single time moment. The correspondence to the avalanche is determined when the cell becomes unstable. The result of the determination can differ from case to case.

### Mega-avalanches and their size

We note that the above dynamics extends the original BTW model with $N = 1$ to the case of $N > 1$. The extension results in several avalanches spreading simultaneously. Resolving this ambiguity, we merge the avalanches that are close in space and time into the mega-avalanches and focus on the probability distribution of the mega-avalanches. A mega-avalanche consists of a single avalanche if this avalanche is not merged with another avalanche.

The proximity between the avalanches is found through the comparison of the Manhattan distance (the sum of the absolute differences of the Cartesian coordinates) $\pmb {\rho }$ with an appropriate function $\phi$ of the avalanches’ sizes. To formalize the rule, we introduce the characteristic (two-state) function ${{\,\mathrm{{\mathbf {1}}}\,}}_{\mathsf {condition}}$ that attains 1 if the $\mathsf {condition}$ holds and 0 otherwise. Let $U\sim \mathbf {Uni}(0,1)$ be a uniform [0, 1] random variable. Then the inequality

4 $$\begin{aligned} {{\,\mathrm{{\mathbf {1}}}\,}}_{\pmb {\rho }(i_1, i_2)< C'L (s_{i_1,t_1}^d + s_{i_2,t_2}^d) } \cdot {{\,\mathrm{{\mathbf {1}}}\,}}_{|t_1 - t_2| \le T} + {{\,\mathrm{{\mathbf {1}}}\,}}_{U < p} \cdot {{\,\mathrm{{\mathbf {1}}}\,}}_{t_1 = t_2} > 0 \end{aligned}$$

underlies the merging of $a_{i_1,t_1}$ and $a_{i_2,t_2}$, where $p \in [0, 1]$, $T \ge 0$, $C' > 0$, and $d > 0$ are the parameters. We fix $C' = 0.025$ and $d = 0.33$, taking them from a range of affordable values. The specific choice affects the other parameters that result in the scale-free distribution of the mega-avalanches.

With $T = 0$ and $p = 0$, (4) becomes

5 $$\begin{aligned} \pmb {\rho }(i_1, i_2) < C'L (s_{i_1,t_1}^d + s_{i_2,t_2}^d), \quad t_1 = t_2. \end{aligned}$$

Therefore, the first term in (4) controls the deterministic merging of the avalanches, specifying a monotone increasing function of sizes $\phi (s_1, s_2) = C'L (s_{i_1,t_1}^d + s_{i_2,t_2}^d)$ that has to exceed the distance $\pmb {\rho }$ between the avalanche origins in order to secure the coalescence. The switch to positive values of *p* admits the random merging of (possibly, small) avalanches located anywhere with the intensity *p*. In general, the second term in (4) means that remote instabilities can occasionally cause one another. Positive integers *T* allow to coalesce the avalanches observed at subsequent time moments. As we will see, a gradual increase in *T* from zero is required rather than the jump to 1. This leads us to the fractional values of $T \in (0, 1)$ and the probabilistic nature of the inequality $|t_1 - t_2| \le T$. This inequality is claimed to hold with certainty if $t_1 = t_2$ and with probability *T* if $|t_1 - t_2| = 1$.

If the avalanches $a_{i_1,t_1}$, $\ldots$, $a_{i_k,t_k}$, $k \ge 1$, form the mega-avalanche *a*, then the size $s = \mathsf {size}(a)$ of *a* is the sum of the corresponding sizes: $s = s_{i_1,t_1} + \ldots + s_{i_k,t_k}$. The origin of the mega-avalanches is the weighted average of the origins of the contributing avalanches, where the weights are proportional to the sizes.

### Probability distribution of the mega-avalanches

**Figure 1:**
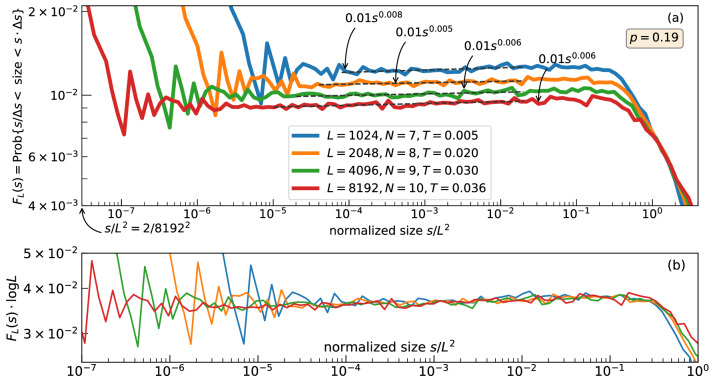
Segment of the size-frequency relation: main result. Power-law segment obtained with exponentially increasing horizontal bins, i. e., the power-law exponent is increased by 1. The vertical axis is not normalized in (**a**) but normalized in (**b**).

Let $f_L(s)$ be the empirical density of the mega-avalanches occurred on the $L \times L$-lattice with respect to their sizes *s* and $F_L(s) = \#\{a: \sigma =\mathsf {size}(a) \in [s / \Delta s, s \Delta s)\} / \#\{a: \sigma =\mathsf {size}(a)> 0\}$ be the proportion of the mega-avalanches with the size located between $s/\Delta s$ and $s \Delta s$, where $\Delta s = 1.2$ is chosen in the graphs (in the continuous case, it would be $f_L(s) = -F_L'(s)$). If $f_L(s)$ follows a power-law $1/x^{\tau }$, so does $F_L(s)$ but the exponent is $\tau -1$ instead of $\tau$. Gathering the points of $f_L(s)$ within the exponentially growing bins into $F_L(s)$, we give the relevant pattern of the power-law segment up to the abrupt bend down in the log-log scale, Fig. 1. The scaling $s \rightarrow s/L^{2}$ normalizes the right endpoint of the power-law segment, Fig. 1.

All four graphs of Fig. 1 (the right part is omitted to highlight the power-law segment) follow an almost flat step corresponding to $s^{1-\tau }$, $\tau \approx 1$, that is turned to a quick decay at the right.

The power-law segments are collapsed after the transformation of the axis: $s \rightarrow s/L^2$, $F_L \rightarrow F_L \log L$ (Fig. 1b). The fact that the transformation $s \longrightarrow s/L^2$ of the horizontal axis normalizing the right endpoint of the power-law segment does not allow to collapse the tails is inherited from the BTW sandpile (because of its multifractal scaling^22^).

**Figure 2:**
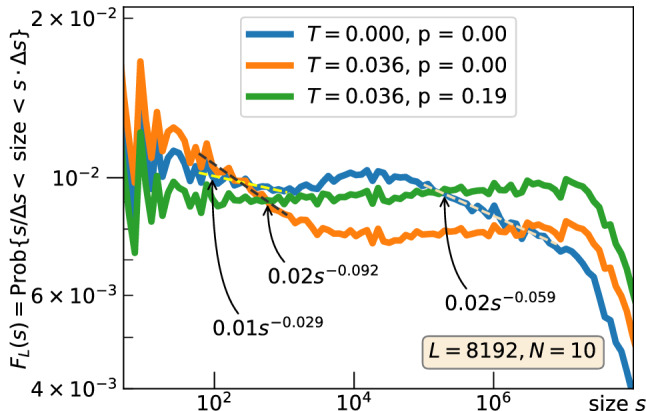
Size-frequency relation: adjustment of parameters. A part of $F_L(s)$ computed with the parameters reported in the legend and $s^{1-\tau }$-fits.

The logarithmic extra-loading $N \sim \log L$ conserves the density of the grains at its critical level (not supported by graphs). The deterministic merging of the avalanches into the mega-avalanches creates two power-law parts of $F_L(s)$ The left part extends to the size of approximately 3000 for all values of *L* (as the blue curve does in Fig. 2), but the right endpoint of second power-law part scales as $L^2$ and its slope becomes steeper as *L* increases (not illustrated here). The introduction of the time clustering with the parameter $T > 0$ makes the right power-law part flatter in the log-log scale (the orange curve in Fig. 2). The contraction of the gap between two consecutive values of *T* shown in Fig. 1 in approximately 1.5 times suggests that *T* saturates at $\approx 0.05$ as $L\rightarrow \infty$.

Interestingly, the changes in the exponent of the right power-law part preserves the existence of the power-law at the left but alters its slope. The return to the flat part of $F_L(s)$ is performed with the random merging through the adjustment of the parameter *p*. The choice of $p=0.19$ is affordable for all graphs constructed with different values of *L*. Thus, our merging is expected to lead to $T\approx 0.05$, $p \approx 0.19$, and $N \sim \log L$ as *L* goes to infinity.

**Figure 3:**
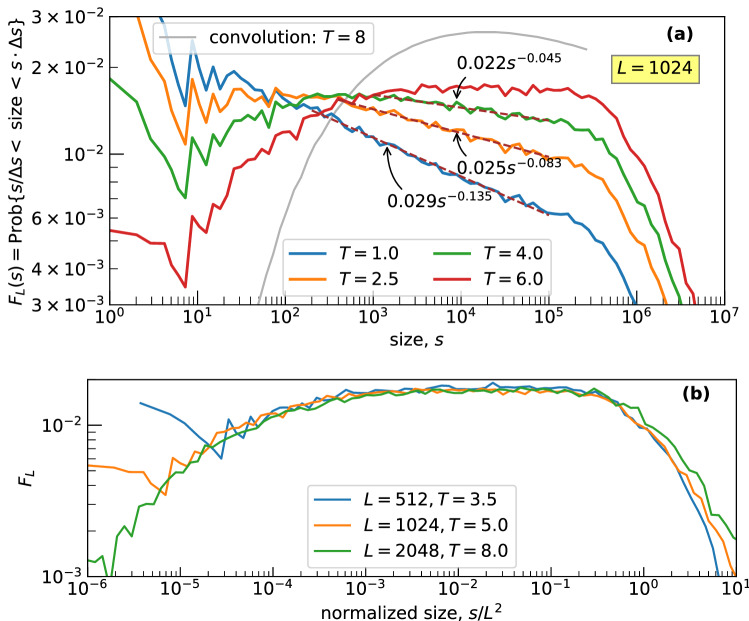
Size-frequency relation $F_L(s)$: BTW model, where the mega-avalanches are obtained through merging of the standard avalanches occurred within *T* consecutive time moments. (**a**) *L* is constant; the gray curve is obtained through the convolution of 8 probability densities $\sim s^{-1.20}$ with the support [1, 1024]. (**b**) *L* is variable; the sizes are normalized by $L^2$ to collapse $F_L(s)$ with adjusted *T*.

## Discussion

We insist that our approach principally differs from the two following simple constructions: the summation of the independent power-law random variables and merging of avalanches, which are adjacent in time, in the original BTW model. The first construction leads to the probability density, which is concave in the log-log scale, tending to the power function at the right part of the graph (the gray curve in Fig. 3 obtained through 8 convolutions, i. e., the summation of 8 independent $1/x^{1.20}$ random variables with the support on [1, 1024]). The second construction can be defined through the coalescence of avalanches occurred during *T* subsequent time moments. The uncertainty with fractional values *T* is resolved with a probabilistic rule (say, if $T = 2.5$ and $a_{t}$ is not merged with $a_{t-1}$, then the avalanches $a_{t}$, $a_{t+1}$, and $a_{t+2}$ are combined with certainty, whereas the avalanche $a_{t+3}$ is added with the probability of 0.5). This modification of the BTW model preserves the power-law segment that does not extend to the right with the growth of the system. The power-law part of $F_L(s)$ constructed for the different values of *L* is collapsed after the normalization of the size by the lattice area, Fig. 3.

Our paper gives evidence that the 1/*x* power-law is feasible with isotropic extensions of the BTW sandpile (Fig. 1). The extension is constructed with the stress accumulation, proportional to $\log L$, and the coalescence of the avalanches propagated closely in space and time. Such a coalescence is known, for example, in seismology, as the stress accumulation and the earthquakes themselves occurred in the slow and quick time respectively are not completely separated^23^. An additional stress accumulation without the construction of mega-avalanches does not lead to the 1/*x* power-law. While the BTW critical density is conserved, the size-frequency relationship of the avalanches (not be confused with the mega-avalanches) follows the power-law segment found with the original BTW sandpile. An excessive loading ruins the critical state, destroying the power-law segment. The construction of the mega-avalanches, performed in the paper with equation (4), are likely to be designed in various ways. Nevertheless, when ignoring spatio-temporal clustering, e.g., assigning $p = 1$ in (4) and merging all avalanches occurred at the same time moment, one also destroys criticality. We argue that spatio-temporal correlations in the BTW-like models, exposed for the BTW sandpile in paper^24^, underlie the possibility to end up with the 1/*x* power-law. Chen et al.^13^ used this correlation, when allowing to pass grains to remote distances with a certain probability $P_c$, and perhaps obtained the power-law exponents that are located above 1.20 and controlled by $P_c$ (the values of the exponents in the thermodynamic limits in their model are not clear as they simulated the model on the $50 \times 50$ lattice). A full description of possibilities, which result in the 1/*x* power-law, and the choice of their “best” version remain the daunting challenge.

Proposed here minor deviations from the BTW model through the parameter domain preserve the critical density of the grains and the power-law size-frequency relationship for the mega-avalanches over the majority of feasible sizes (Fig. 2). The adjustment of the parameters pulls the exponent $\tau$ towards 1 (through a weak time clustering, parameter *T*) and corrects the slope of the restricted left part to fit the whole power-law segment (with the random coalescence in space, parameter *p*). Thus, our approach does not require any tuning of the dissipation-to-loading ratio as in attempts to relate self-organized criticality to the phase transition modeling^25^ but controls the universality class of the sandpile and might lead to adjustable power-law exponents in a neighborhood of 1. Furthermore, the horizon of the avalanche grouping in time, given by *T*, acts as the level of noise in the system that (see arguments of paper^26^) increases the power-law exponent. Eventually, the BTW-like sandpile with the control of the power-law exponent would improve our understating of real-life SOC-phenomena.

## Methods

We have sampled the data for the empirical functions $f_L(s)$ and $F_L(s)$ for $5 \cdot 10^5$ subsequent time moments for all lattices. Sampling is performed after some transient period to let the system reach the steady state and eliminate the dependence on the initial conditions.

**Figure 4:**
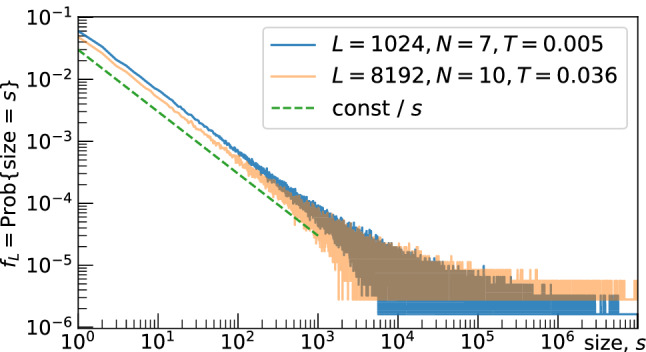
The probability density function $f_L(s)$ of mega-avalanche’s sizes; the part with $s > 10^7$ is omitted.

The summation of the probability density $f_L(s)$ over exponentially increasing bins increases the exponent of the power-law from $-\tau$ to $-\tau +1$, as the sum follows the integral:

$$\begin{aligned} \int _{s/\Delta s}^{s\Delta s} \pmb {\sigma ^{-\tau }}\,d\sigma = {\left\{ \begin{array}{ll} \frac{1}{1-\tau } \big ( (\Delta s)^{1-\tau } - (\Delta s)^{\tau -1} \big ) \cdot \pmb {s^{1-\tau }}, &{} \text {if}\, \tau \ne 1, \\ 2\ln \Delta s \cdot \pmb {s^0}, &{} \text {if}\, \tau = 1 \end{array}\right. } \end{aligned}$$

as *s* is large. In contrast to $F_L(s)$, the graph of $f_L(s)$ is too noisy at the right to illustrate the full power-law segment (Fig. 4 exhibits $f_L(s)$ found with $L=1024$ and $L=8192$).

The logarithmic correction of the vertical axis required for the collapse of the power-laws in Fig. 1b is caused by the proximity of the probability density to 1/*s*-segment, the power-law scaling of the right endpoint $s^*$ of this segment, and a fast decay of $F_L$ at the right from $s^*$. Then the integration of the density $f_L(s) = C_L / s$ over $[1, +\infty ]$ results in the estimate $C_L \cdot c \log L \approx 1$, which implies $C_L \sim 1 / \log L$.
